# Increased frequency of rare damaging variants in *PLIN4* in patients with anorexia nervosa

**DOI:** 10.3389/fpsyt.2026.1937613

**Published:** 2026-10-08

**Authors:** Michael Lutter, Lucas G. Casten, Jacob J. Michaelson, Guido K. W. Frank, Alenka Čopič, Michael S. Mazei-Robison, Alfred J. Robison

**Affiliations:** 1 Precision Psychiatry, Plano, TX, United States; 2 Department of Psychiatry, University of Iowa, Iowa City, IA, United States; 3 Department of Psychiatry, University of California, San Diego, San Diego, CA, United States; 4 Centre de Recherche en Biologie Cellulaire de Montpellier (CRBM), Universite de Montpellier, French National Center for Scientific Research (CNRS), Montpellier, France; 5 Department of Physiology, Michigan State University, East Lansing, MI, United States

**Keywords:** anorexia nervosa, eating disorder, lipid droplet, perilipin 4, whole exome sequencing

## Abstract

**Background:**

Anorexia nervosa (AN) is a severe psychiatric disorder characterized by pathological fear of weight gain, persistent restriction of energy intake, and pervasive body image disturbance. Although AN is substantially heritable, the rare coding variants contributing to susceptibility remain incompletely defined. Pedigree-based whole-exome sequencing studies can help to identify rare, high-effect protein-coding variants and thereby elucidate disease mechanisms.

**Methods:**

Whole-exome sequencing was performed on AN-dense family pedigrees to identify candidate genes. The frequency of rare (minor allele frequency <1%), putatively damaging [combined annotation-dependent depletion (CADD) >15, including predicted loss-of-function (LOF)] variants in 32 candidate genes was assessed in 154 unrelated probands of European ancestry. Carrier counts were compared against gnomAD v4 non-Finnish European reference using a TRAPD-style coverage-adjusted denominator (maximum allele number/2 across qualifying sites per gene). Significance was assessed by one-sided Fisher exact test, Bonferroni-corrected across 32 candidate genes.

**Results:**

*PLIN4* was the only gene enriched in AN cases at Bonferroni-corrected significance: 17 of 154 patients (11.0%) carried a rare damaging variant versus 3.2% in gnomAD non-Finnish Europeans [odds ratio (OR) 3.86, 95% confidence interval (CI) 2.35 to 6.35; one-sided Fisher *p* = 9.98e−06; Bonferroni-corrected *p* = 3.19e−04]. The result is preserved with the index proband removed (*n* = 153; OR 3.64, 95% CI 2.18 to 6.07; corrected *p* = 1.13e−03). Six of 154 patients carried predicted LOF variants; 4 of 6 had at least one AN-affected first- or second-degree relative carrying the same variant, providing co-segregation evidence. The LOF-only burden did not survive Bonferroni correction in isolation.

**Conclusions:**

Patients with AN carry a statistically significant excess of rare, putatively damaging variants in *PLIN4*, at a threshold surviving Bonferroni correction across 32 candidate genes. These findings implicate lipid-droplet biology as a candidate contributor to AN pathophysiology.

## Introduction

Anorexia nervosa (AN) is a severe psychiatric disorder characterized by pathological fear of weight gain, persistent restriction of energy intake, and pervasive body image disturbance. Affected individuals may restrict caloric intake, engage in purging behaviors (including self-induced vomiting), and exercise compulsively to avoid weight gain, often progressing to life-threatening emaciation. Although certain psychotherapies improve short-term weight restoration in adolescents (1), a substantial proportion of patients derive no lasting benefit from currently available psychotherapeutic (2) or pharmacological (3) interventions.

AN risk arises from the interplay of environmental and genetic factors. Susceptibility variants have been identified through genome-wide association studies (GWAS) (4) and pedigree-based family segregation studies (5). These approaches are complementary: GWAS identify common variants of individually modest effect, but identified loci typically reside in non-coding regulatory regions, limiting mechanistic inference (4). Family segregation studies, by contrast, analyze multi-generation pedigrees to identify rare, high-effect variants. Because these variants often reside within protein-coding regions, their functional consequences are more readily characterized. For example, a pedigree study identified rare coding variants in *ESRRA* and *HDAC4* in two families with multiple AN-affected members (5). These findings enabled the development of two monogenic mouse models of disordered eating behavior (6, 7), which were subsequently used to characterize biochemical and electrophysiological perturbations that may underlie the observed behavioral changes (7, 8).

While a GWAS had identified several common-variant risk loci for AN (4), large-scale rare-variant studies in AN remain limited. Walker et al. (9) reported the largest copy number variant (CNV) burden analysis to date (7,414 AN cases) and found no significant enrichment of rare CNVs, positioning rare point-mutation burden studies as the primary discovery paradigm for AN rare-variant biology. Similar discovery strategies have yielded translational models implicating lipid metabolism genes (10, 11), brain-derived neurotrophic factor (*BDNF*) (12), and peptide neurotransmitter genes (13–16).

Understanding the full genetic architecture of AN therefore requires characterization of both common and rare variant contributions. Here, we report that predicted loss-of-function (LOF) and putatively damaging [combined annotation-dependent depletion (CADD) > 15] variants in *PLIN4* are significantly enriched in patients with AN relative to population controls. *PLIN4* encodes perilipin 4, a member of the perilipin family of lipid-droplet (LD) coat proteins (17–19). No analogous enrichment was observed for other perilipin genes (*PLIN1*, *PLIN2*, *PLIN3*, or *PLIN5)*, suggesting a *PLIN4*-specific contribution to AN susceptibility.

## Materials and methods

### Participants and clinical assessment

The study cohort comprised 154 unrelated individuals who presented to Precision Psychiatry (Plano, TX) for evaluation of AN. Whole-exome sequencing (WES) was offered to patients meeting one of three criteria: (i) a strong family history of eating disorders (at least three affected family members); (ii) multiple failed courses of evidence-based treatment; or (iii) an atypical clinical presentation or unusual laboratory findings raising suspicion of a rare inherited metabolic disorder, such as mitochondrial disease (20), medium-chain acyl-CoA dehydrogenase deficiency (21), or citrin deficiency (22). All participants provided written informed consent for use of genetic findings for clinical and research purposes. The cohort was predominantly female assigned at birth (148/154; 96.1%) and of non-Finnish European (NFE) ancestry (150/154; 97.4%). In gnomAD, the NFE stratum excludes Finnish-ancestry samples because Finnish founder effects generate population-specific allele frequencies that would bias rare-variant comparisons (23). Cohort-level demographics are summarized in **Table 1**.

**Table 1:** Cohort demographics (*n* = 154).

Characteristic	*n* (%) or median (IQR)
Sex	
Female assigned at birth	148 (96.1)
Ancestry	
Non-Finnish European	150 (97.4)
AN subtype	
Restrictive	104 (67.5)
Binge-purge	37 (24.0)
Atypical	13 (8.4)
Age at onset, years*	15.8 (14.2–16.9)
Nadir BMI, kg/m²*	14.9 (13.0–16.1)

AN, anorexia nervosa; BMI, body mass index; IQR, interquartile range. *Age at onset and Nadir BMI reported as median (IQR). Age at onset missing for six patients (*n* = 148 with available data).

*Median (IQR); age at onset missing for six patients (recorded as N/A).

### Cohort lineage

The 154 unrelated patients with AN in the present analysis were recruited under the Precision Psychiatry clinic (Plano, Texas) Institutional Review Board (IRB)-approved protocol (International Federation of Eating Disorder Dietitians Institutional Review Board, HHS Registration Number IRB00013903, IRB Tracking Number E-2025-0001), with informed consent for genetic analysis. The same recruitment pipeline, IRB protocol, and WES pipeline (Novogene 100×; BWA-MEM to GRCh38/hg38; GATK joint genotyping; ANNOVAR; CADD v1.6 pathogenicity priors) underpin both the present analysis and the previously reported *BBOX1* burden cohort (*n* = 183) (11).

The *PLIN4* cohort (*n* = 154) is a complete subset of the *BBOX1* cohort. All 154 patients in the *PLIN4* analysis are included within the 183-patient *BBOX1* cohort; the 29 patients present only in the *BBOX1* cohort were excluded from the *PLIN4* analysis because of insufficient depth of sequencing coverage within the *PLIN4* gene to permit accurate variant calls. The union of the two cohorts therefore comprises 183 unique unrelated patients with AN.

The combined *PLIN4* + *BBOX1* carrier statistics reported in the Discussion section are computed on the 183 unique unrelated patients with AN comprising this union cohort.

The cohort is a clinically ascertained referral population from a specialist eating-disorders clinic and is not a population sample of AN. Carrier-frequency and burden-enrichment estimates may therefore differ from those expected in a population-ascertained cohort. This ascertainment is addressed as a pre-specified limitation in the Discussion section.

### Whole-exome sequencing and variant calling

Buccal swabs were collected using iSWAB-Discovery (#ISAWB-DSC, Mawi DNA Technologies LLC, Pleasanton, CA). Samples were submitted to Novogene (Sacramento, CA) for clinical 100× WES (Agilent SureSelect V6–60 M, Agilent, Santa Clara, CA; ABclonal Rapid Plus DNA Lib Prep Kit, ABclonal, Woburn, MA). Mean sequencing depth was 169.04×, with at least 20× coverage across 97.4% of targeted exons. Briefly, genomic DNA was randomly sheared into fragments of 180–280 base pairs, end-repaired, A-tailed, and ligated to Illumina adapters. Adapter-ligated fragments were PCR-amplified, size-selected, and purified. Hybridization capture used biotin-labelled probes with streptavidin-conjugated magnetic bead precipitation to isolate target exons; non-hybridized fragments were removed and probes were digested. Captured libraries were PCR-amplified, quantified by Qubit and real-time PCR, and sequenced on Illumina platforms using the PE150 protocol.

Bioinformatic analysis included the following: (i) data quality control by filtering reads containing adapters or with low quality (fastp v0.23.1); (ii) alignment to the human reference genome GRCh38/hg38 with BWA v0.7.17; (iii) duplicate marking with Sambamba v1.0.0 and merging with Picard v2.18.9; and (iv) germline variant detection using GATK v4.3.0 HaplotypeCaller (24) for single nucleotide polymorphisms (SNPs) and small insertions and deletions (<50 bp; InDels). The GATK VariantFiltration module was applied with standard hard-filter parameters. Variants were annotated using ANNOVAR (25), including protein-coding changes, genomic regions, allele frequency, and pathogenicity prediction.

Of the 154 unrelated probands in the WES cohort, the index family was selected for pedigree-based candidate-gene discovery because it had the highest density of AN-affected first- and second-degree relatives at the time of analysis. No other family in the cohort had more than two AN-affected relatives reported at the time of pedigree selection.

### gnomAD comparison and denominator definition

Reference allele counts came from gnomAD v4 NFE exomes (9, 23, 26). For each gene, the case numerator was the count of AN carriers of a rare damaging variant (CADD > 15 or predicted LOF). The control numerator was the per-gene sum of AC minus twice the sum of homozygote counts across qualifying sites (heterozygous carrier alleles in NFE controls).

The control denominator was max(AN)/2 per qualifying site, where AN is the gnomAD v4 NFE allele number. This canonical TRAPD approach (27) is maximally conservative, inflating the denominator and yielding a lower-bound odds ratio (OR) estimate. Empirical coverage across the 32 candidate genes ranged from 89.9% to 94.8% of the gnomAD v4 NFE exome total (median 94.2%), consistent with adequate power for burden comparisons.

Re-running with the median(AN)/2 denominator yielded results within 1% of the primary analysis (**Supplementary Table S2**).

The Phred-scaled CADD score was selected as the primary pathogenicity prior because it has similar specificity for identifying damaging variants to that of other common methods, such as SIFT and MutationTaster2, and allows for an easily interpretable unit for external comparison (28). A score above 10 indicates that a variant is in the top 10% of most deleterious variants, a score above 20 indicates the top 1%, and a score of 30 or greater indicates the top 0.1% (29).

### Multiple-testing correction and significance thresholds

The candidate-gene set comprised the 32 genes carrying rare CADD > 15 or LOF variants shared in the index pedigree (31 unique gene symbols; **Supplementary Table S1**). Enrichment was tested per gene using one-sided Fisher exact tests (alternative = greater), the pre-specified direction for case-versus-population burden hypotheses (27). Family-wise error rate was controlled by Bonferroni correction across the 32 candidate genes. A secondary threshold using a denominator of 44—the full pedigree-screen variant count including non-CADD-prioritized entries—is reported alongside. Two-sided Fisher exact tests were additionally computed to identify genes significantly depleted of rare damaging variants relative to gnomAD. The modern reimplementation of TRAPD (30) was the methodological reference for case-versus-gnomAD burden testing; the original TRAPD GitHub repository is no longer maintained. Alternative set-based rare-variant association methods (31) require individual-level genotype data in controls and were therefore not applicable to the present gnomAD-summary-statistic design.

### Statistical analysis

All burden tests, ORs, and confidence intervals (CIs) were computed in R. ORs used the Haldane–Anscombe correction (+0.5 to all four cells); 95% CIs were derived from the Wald log-OR standard error. Fisher exact tests (one-sided and two-sided) were performed with the fisher.test() function. Multiple-testing correction was applied per the “Multiple-testing correction and significance thresholds” section. All numerical results trace to the per-gene burden table (**Table 2**).

**Table 2:** Thirty-two-gene burden, Bonferroni-corrected across 32 candidate genes (*n* = 153 primary, *n* = 154 sensitivity).

Primary analysis: proband removed (*n* = 153 AN cases)						
Gene	AN cases with rare variants/n (153)	gnomAD v4 NFE carriers/denominator*	OR (95% CI)	*p*-value (one-sided)	Bonferroni *p*	Direction
*PLIN4*	16/153	18,724/568,226	3.64 (2.18–6.07)	3.52 × 10^-5^	1.13 × 10^-3^	Enriched
*KIAA0754*	13/153	107,226/474,871	0.43 (0.24–0.74)	0.9998	1.00†	Depleted

Sensitivity analysis: proband retained (*n* = 154 AN cases)						
Gene	AN cases with rare variants/n (154)	gnomAD v4 NFE carriers/denominator*	OR (95% CI)	*p*-value (one-sided)	Bonferroni *p*	Direction
*PLIN4*	17/154	18,724/568,226	3.86 (2.35–6.35)	9.98 × 10^-6^	3.19 × 10^-4^	Enriched
*KIAA0754*	13/154	107,226/474,871	0.42 (0.24–0.74)	0.9998	1.00†	Depleted

OR, odds ratio; CI, confidence interval; NFE, non-Finnish European; gnomAD, Genome Aggregation Database; LOF, loss-of-function; CADD, combined annotation-dependent depletion. Rare variants defined as CADD > 15 (putatively damaging) or predicted LOF. OR uses Haldane–Anscombe continuity correction (+0.5 to all four cells); 95% CI from Wald log-OR standard error. *gnomAD v4 NFE denominator = per-gene representative AN/2 from gnomAD v4 NFE exomes (PLIN4: 568,226; KIAA0754: 474,871). ^†^KIAA0754 signal is in the depleted direction; not interpreted as a risk locus. Full 32-gene results are provided in **Supplementary Table S2**.

Counts: a = AN carriers; b = AN non-carriers; c = gnomAD v4 NFE carrier alleles (sum AC minus 2x homozygote count, aggregated across CADD > 15 and LOF variant sites); d = gnomAD v4 NFE non-carrier individuals (representative AN/2 across variant sites in the gene, minus c). OR uses Haldane–Anscombe correction (+0.5 to all four cells); 95% CI from Wald log-OR standard error.

Full cohort (*n* = 154, including proband). Carrier count (*n* = 17) includes the proband; primary burden analysis (**Table 2**) used proband-removed cohort (*n* = 153, 16 carriers).

## Results

### Index family pedigree

The proband for the index family presented with treatment-resistant AN and was offered WES on the basis of a strongly positive family history: her mother and two sisters all carried AN diagnoses (**Figure 1**). The index family was selected for pedigree-based candidate-gene discovery because it had the highest density of AN-affected first- and second-degree relatives in the cohort at the time of analysis. Analysis was restricted to rare (MAF < 0.01) coding variants shared across all affected family members. WES identified 44 such coding variants (**Supplementary Table S1**); *in silico* pathogenicity scoring prioritized 32 (CADD > 15) as putatively damaging, of which 2 were predicted LOF. Variants with the highest CADD scores mapped to *SYNC*, *CDK15*, *MAS1*, *CSMD3*, *GRIK4*, *HAL*, *EAPP*, and *PLIN4*. While none of the harboring genes had previously been associated with AN risk or regulation of feeding behavior, the rs8887 variant in *PLIN4* has previously been linked to enhanced sensitivity to dietary omega-3 polyunsaturated fatty acids (32). The PLIN4 protein also has an established role in cellular lipid storage and whole body fat distribution (33–35), an anthropometric trait with well-characterized genetic correlates in AN (4). Therefore, *PLIN4* was selected for follow-up based on potential involvement in physiological processes relevant to the development of AN.

**Figure 1:**
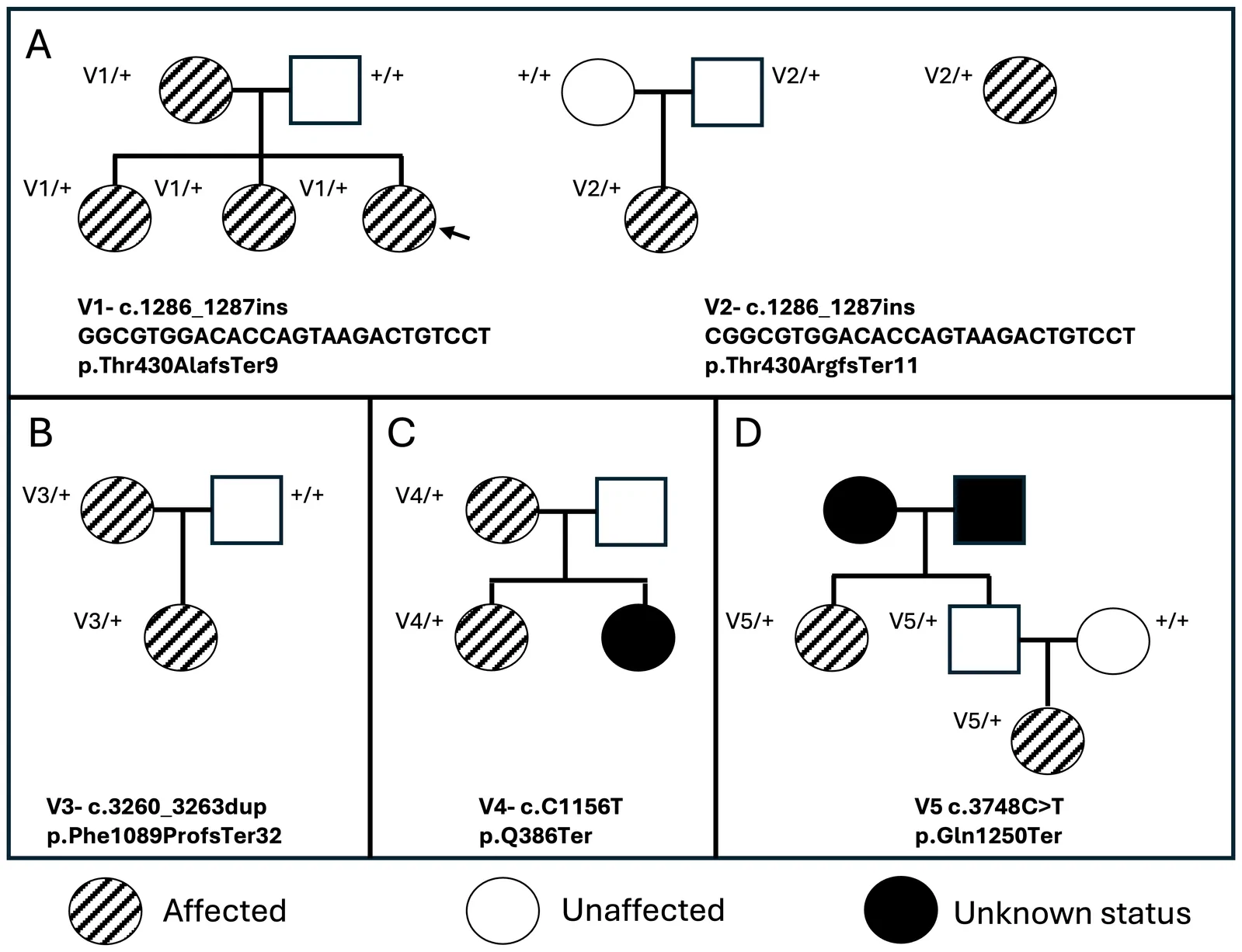
Pedigree of the index family with multiple AN-affected relatives carrying a *PLIN4* LOF variant (rs780147097), and pedigrees of additional unrelated individuals carrying distinct *PLIN4* LOF variants with affected first- or second-degree relatives sharing the same variant. Panels **(A–D)** show the familial co-segregation. Genetic variant labels (V1–V5) denote HGVS transcript and protein nomenclature for *PLIN4* (NM_001367868) with + representing wild type. Each pedigree symbol is labeled with the individual genotype next to it; squares = male; circles = female; arrow = index proband. All participants provided informed consent; pedigrees are presented in anonymized schematic form.

### *PLIN4* variants in the unrelated AN cohort

To evaluate the cohort-wide burden of *PLIN4* LOF alleles, all 154 unrelated probands were screened for predicted LOF variants. The rs780147097 variant is multiallelic: while no additional proband carried the same 26-base-pair insertion, two further probands harbored a 32-base-pair insertion at the same chromosomal position (**Figure 1**; **Supplementary Table S1**). Three additional unrelated probands each carried a distinct predicted LOF variant elsewhere in *PLIN4* (**Figures 1B–D**); in each case, a female first- or second-degree relative (one paternal aunt, one mother, and one daughter, respectively) carried the identical LOF allele and had a confirmed AN diagnosis. In total, 6 of 154 unrelated AN probands (3.9%) and 6 of their female relatives carried a predicted *PLIN4* LOF variant.

We subsequently screened all probands for rare (MAF < 0.01), putatively damaging (CADD > 15) *PLIN4* variants. An additional 11 probands carried seven distinct putatively damaging single nucleotide variants in *PLIN4* (**Supplementary Table S1**). The combined observed frequency of 17/154 (11.0%) was compared with the rate in gnomAD v4 NFE individuals (3.2%), revealing a significantly elevated burden of putatively damaging *PLIN4* variants among patients with AN (OR 3.86, 95% CI 2.35 to 6.35; one-sided Fisher *p* = 9.98e−06). No other member of the perilipin gene family (19) showed significant enrichment of rare damaging variants among AN cases.

### Burden analysis of 32 genes with multiple-testing correction

To address the *post-hoc* nature of *PLIN4* selection, all 32 candidate genes from the index pedigree were tested for burden against gnomAD v4 NFE controls using one-sided Fisher exact tests, with Bonferroni correction across 32 tests (**Table 2**). Of the 32 candidate genes, only *PLIN4* reached Bonferroni-corrected significance in the enriched direction. In the primary framing (proband retained, *n* = 154), 17 patients with AN carried a rare damaging *PLIN4* variant versus a gnomAD NFE carrier rate of 3.2% (OR 3.86, 95% CI 2.35 to 6.35; one-sided Fisher *p* = 9.98e−06; Bonferroni-corrected *p* = 3.19e−04). In the internal-replication framing (index proband removed, *n* = 153), 16 patients with AN carried a qualifying variant (OR 3.64, 95% CI 2.18 to 6.07; one-sided Fisher *p* = 3.52e−05; Bonferroni-corrected *p* = 1.13e−03).

The *PLIN4* burden is consistent across both primary and internal-replication framings. *KIAA0754* was the only other gene reaching Bonferroni-corrected significance across the 32 candidates, in the depleted direction (*n* = 153: 13/153 carriers, OR 0.43, 95% CI 0.24 to 0.74, two-sided Fisher *p* = 7.88e−04, Bonferroni-corrected *p* = 0.025; *n* = 154: OR 0.42, two-sided Fisher *p* = 7.93e−04, Bonferroni-corrected *p* = 0.025). Depletion is not consistent with AN risk elevation and likely reflects a high-frequency benign variant inflating the gnomAD control denominator for this gene. No other gene reached Bonferroni-corrected significance in either direction. A forest plot of the 10 genes with the smallest one-sided Fisher exact *p*-values is presented in **Figure 2**.

**Figure 2:**
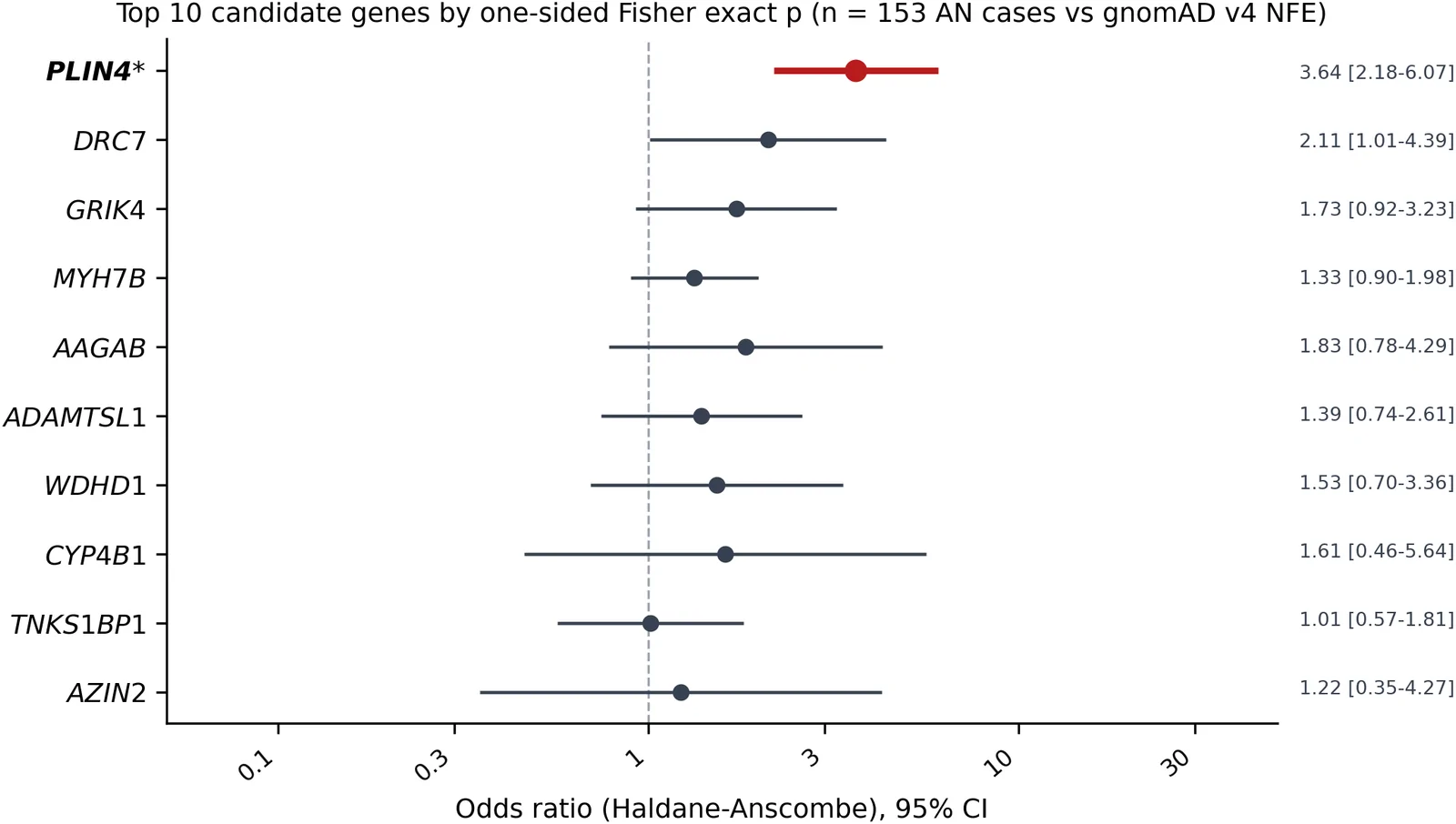
Forest plot of the 10 candidate genes with the smallest one-sided Fisher exact *p*-values for combined CADD > 15 + LOF rare-variant burden in *n* = 153 AN cases (proband removed) versus gnomAD v4 non-Finnish European controls. Points show the Haldane–Anscombe corrected OR; whiskers show the 95% Wald CI (truncated where the upper bound exceeds 50). The dashed vertical line marks OR = 1. *PLIN4* (bold) is the only gene that survives Bonferroni correction across all 32 candidate genes in the enriched direction. *KIAA0754* also crosses Bonferroni correction but in the depleted direction (two-sided); see **Table 2** for the full 32-gene listing. *Bonferroni-corrected one-sided Fisher exact *p* < 0.05 across 32 candidate genes.

### LOF-only sensitivity analysis

LOF-only burden in *PLIN4* is reported separately from the combined CADD > 15 + LOF burden. In the *n* = 154 unrelated cohort, 6 patients carried a *PLIN4* LOF variant; in the proband-removed *n* = 153 framing, 5 patients carried a LOF variant. LOF-only burden statistics: *n* = 154 framing, OR 4.56 (95% CI 2.08 to 10.01), one-sided Fisher *p* = 3.8e−03, Bonferroni-corrected *p* = 0.12; *n* = 153 framing, OR 3.86 (95% CI 1.65 to 9.04), one-sided Fisher *p* = 0.016, Bonferroni-corrected *p* = 0.51. Under either framing, LOF-only burden does not survive Bonferroni correction across the candidate-gene set. The multiple-testing-survived statistical signal is therefore the combined CADD > 15 + LOF burden, not LOF alone.

LOF carriers nevertheless contribute biologically informative co-segregation evidence: four of the six unrelated LOF carriers in the *n* = 154 cohort have at least one female relative with a history of AN or avoidant/restrictive food intake disorder (ARFID) carrying the same *PLIN4* LOF variant, providing within-family evidence of pathogenicity across multiple independent families. Among the four co-segregating families: the index proband’s mother and two sisters (first-degree relatives) carry rs780147097; one additional proband had a paternal aunt (second-degree relative) carrying the same LOF variant; one proband had her mother (first-degree) carrying the same LOF variant; and one proband had her daughter (first-degree) carrying the same LOF variant. Full per-family variant assignments are provided in **Supplementary Table S1**.

The pedigree-level and cohort-level LOF counts are distinct and are not interchanged. The index pedigree carries *PLIN4* LOF variants distributed across six family members of the proband (pedigree-level). The unrelated cohort search identified six LOF carriers in the *n* = 154 analysis (cohort-level: one proband + five additional unrelated patients). These two numbers describe different analytic units and are reported separately throughout.

#### EAPP co-carriage disclosure

The index proband is a co-carrier of LOF variants in both *PLIN4* and *EAPP* (rs370344686; p.Glu19Ter). We disclose this co-carriage transparently because it bears on variant-level reporting and because the proband contributes to the *n* = 154 framing for both genes. The *EAPP* LOF is reported in **Supplementary Table S1** for completeness; *EAPP* burden is not significant after Bonferroni correction across 32 candidates (1/154 carriers, OR 0.98 in the *n* = 154 framing; 0/153 carriers, OR 0.33 in the *n* = 153 framing) and is not interpreted as an independent AN-risk locus. The *PLIN4* finding is independent of *EAPP* co-carriage; *PLIN4* burden remains Bonferroni-significant after exclusion of the proband (*n* = 153 framing).

## Discussion

Patients with AN exhibit a characteristic fear of gaining weight and an aversion to dietary fat intake (36). In the present study, we report that patients with AN carry a significantly elevated burden of rare, putatively damaging variants in *PLIN4*, a gene encoding the LD coat protein perilipin 4 (**Figure 3**). Under positive energy balance, excess fatty acids are esterified to triacylglycerides (TAG) and sequestered within cytosolic LD, buffering cells against lipotoxicity (19). Members of the perilipin family (*PLIN1* - 5) are principal structural constituents of LD, stabilizing the lipid-aqueous interface and regulating TAG accessibility to lipases. Under energy deficit, adrenergic signaling promotes lipase recruitment to the LD surface, initiating TAG hydrolysis and release of fatty acids that are conjugated to L-carnitine for import into the mitochondrial matrix via the carnitine palmitoyltransferase system for beta-oxidation (19).

**Figure 3:**
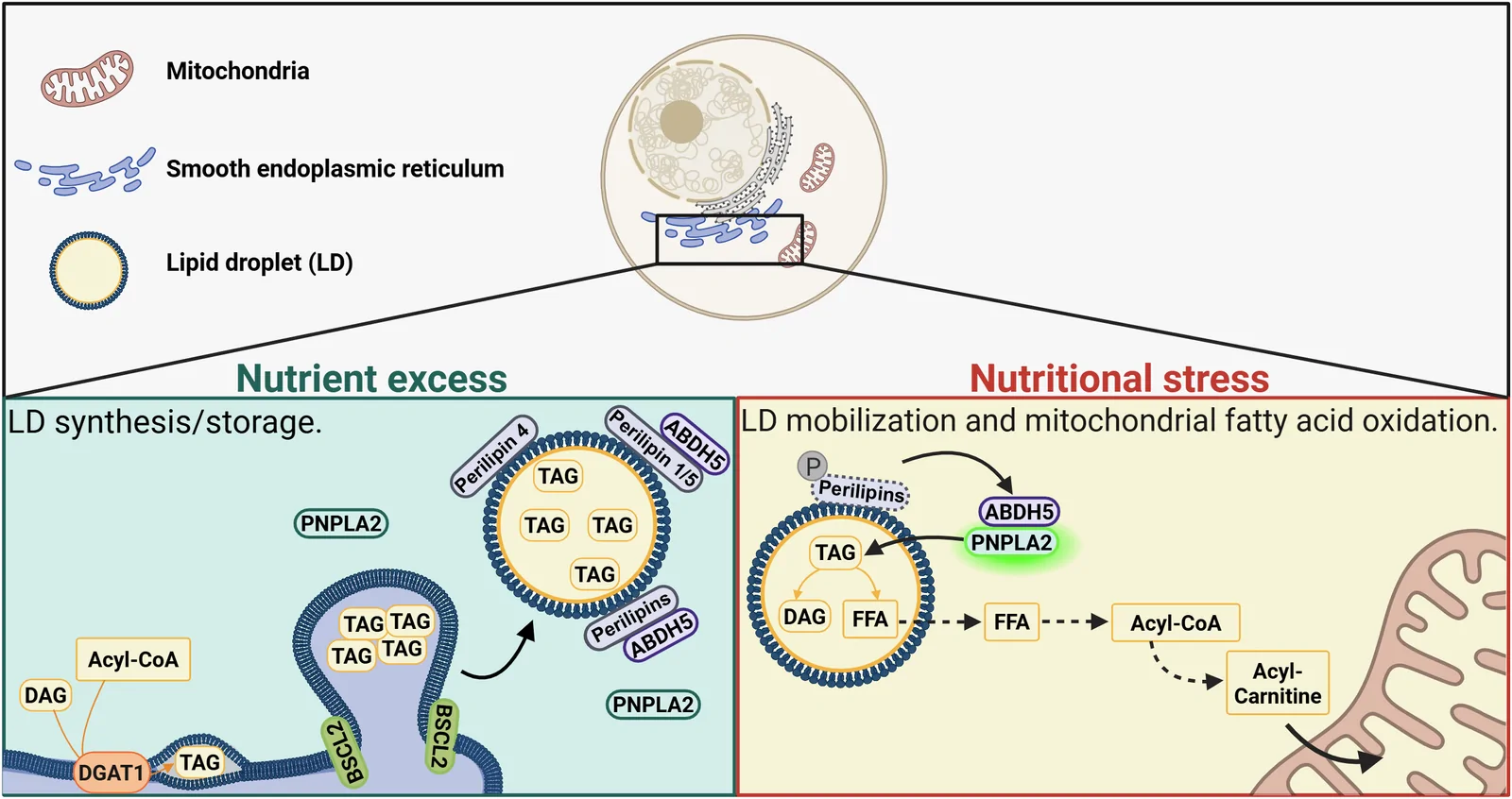
Schematic of perilipin-mediated lipid-droplet (LD) biology in adipocytes and skeletal muscle. Under conditions of nutrient excess (left panel), diacylglycerol (DAG) is esterified to triacylglycerol (TAG) by DGAT1, packaged into nascent LDs, and stabilized by perilipin coat proteins including PLIN4. Under nutritional stress (right panel), ATGL/PNPLA2 hydrolyses TAG to free fatty acids (FFA) via DAG intermediates; FFAs are activated to acyl-CoA and imported into the mitochondrial matrix as acyl-carnitine for beta-oxidation. Predicted loss-of-function variants in PLIN4 are hypothesized to impair both LD homeostasis, contributing to the dyslipidemia and altered body fat distribution observed in AN carriers.

### Significance threshold and multiple-testing context

The reported burden *p*-value (9.98e−06 in the *n* = 154 framing; 3.52e−05 in the *n* = 153 internal-replication framing) does not meet the genome-wide significance threshold of 5e−08 used in unbiased GWAS scans. However, the present analysis is a pedigree-anchored candidate-gene burden test rather than a hypothesis-free genome-wide scan; the appropriate multiple-testing yardstick is Bonferroni correction for the *n* = 32 candidates from the initial pedigree screen (corrected *p* = 3.19e−04 in the *n* = 154 framing; 1.13e−03 in the *n* = 153 framing), which the result survives. Genome-wide significance thresholds are calibrated for the approximately 10^6^ effectively independent common variants tested in GWAS, not for a pre-specified candidate-gene panel.

### LOF carriers: biological co-segregation evidence

The LOF carriers in our cohort (*n* = 6 in the *n* = 154 cohort search; 1 proband + 5 additional unrelated patients) constitute the primary biological and co-segregation evidence: four of six carriers have AN-affected first- or second-degree relatives carrying the same LOF variant, demonstrating co-segregation across multiple independent families. However, the LOF-only burden does not survive Bonferroni correction across the 32 candidate genes. The statistical signal that survives multiple-testing correction is the combined CADD > 15 + LOF burden test. We interpret these two analyses as complementary: the LOF carriers carry the family-level co-segregation evidence that motivates a biological model, and the combined burden test carries the multiple-testing-survived statistical signal across the unrelated cohort. Both pieces of evidence are necessary; neither is sufficient alone.

### Biological context: *PLIN4*, lipid droplets, and AN phenotypes

Recently, we reported an elevated burden of rare, putatively damaging variants in *BBOX1* among patients with AN (11). *BBOX1* encodes an enzyme in the L-carnitine biosynthesis pathway; L-carnitine is required for import of long-chain fatty acids from LDs into the mitochondrial matrix for beta-oxidation. The *PLIN4* cohort (*n* = 154) is a complete subset of the *BBOX1* cohort (*n* = 183); the union comprises 183 unique unrelated patients with AN. Of these, 16 carry a rare damaging variant in *PLIN4* only, 11 carry a rare damaging variant in *BBOX1* only, and 1 carries damaging variants in both genes, for a combined carrier rate of 28/183 (15.3%). If confirmed in independent cohorts, these observations implicate impairment of fatty acid storage and mitochondrial import as a shared molecular mechanism predisposing to AN (**Figure 3**).

First, genetic studies have linked predicted LOF variants in *PLIN4* with differences in body weight, body fat percentage, and waist–hip ratio (33, 34, 37), consistent with the genetic correlations between AN and these anthropometric measures identified in GWAS (4). Notably, individuals carrying *PLIN4* LOF variants show selectively reduced gynoid fat depots (hips, thighs, and buttocks) with reciprocally elevated trunk fat accumulation (33). This pattern is directly relevant to AN with multiple independent studies demonstrating that weight restoration in adult patients with AN disproportionately accrues as truncal adiposity (38–41).

Second, impaired LD function may underlie the hypertriglyceridemia observed in patients with AN even after weight restoration (42). Predicted *PLIN4* LOF variants are independently associated with elevated plasma triglycerides (33). Consistent with this, five of the six unrelated *PLIN4* LOF carriers in the present cohort had a documented clinical history of hypertriglyceridemia or hyperlipidemia; the remaining carrier had a concurrent diagnosis of lymphocytic colitis, a malabsorptive condition that may attenuate the hyperlipidemia phenotype. Across the full carrier group (LOF plus CADD > 15 damaging variants), 8 of 17 *PLIN4* carriers (47.1%) had a clinical diagnosis of hyperlipidemia, compared with 11.7% (16/137) of non-carriers (**Table 3**, OR 6.72, 95% CI 2.09-21.55; Fisher two-sided *p* = 0.0011). Although these comparisons are derived from clinical records rather than standardized lipid panels, and the carrier sample is modest in size, the direction and magnitude are consistent with the established association of *PLIN4* LOF with elevated triglycerides (33) and provide direct clinical corroboration of the lipid-biology model.

**Table 3:** Descriptive characteristics of *PLIN4* carriers vs. non-carriers.

Characteristic	PLIN4 carriers (*n* = 17)	Non-carriers (*n* = 137)	*p*-value
Variant class			
Predicted LOF, *n* (%)	6 (35.3)	0	–
Damaging missense (CADD > 15), *n* (%)	11 (64.7)	0	–
AN subtype, *n* (%)			
Restrictive	11 (64.7)	93 (67.9)	0.79
Binge-purge	5 (29.4)	32 (23.4)	0.57
Atypical	1 (5.9)	12 (8.8)	1.00
Clinical measures			
Age at onset, years*	14.6 (13.0–15.8)	16.0 (14.2–17.0)	0.047
Nadir BMI, kg/m²*	14.0 (11.0–15.0)	15.0 (13.0–16.6)	0.051
Psychiatric comorbidities, *n* (%)			
Mood disorder (MDD or bipolar)	16 (94.1)	121 (88.3)	0.70
Anxiety disorder	17 (100)	133 (97.1)	1.00
OCD	6 (35.3)	55 (40.1)	0.80
ADHD	2 (11.8)	29 (21.2)	0.52
Medical comorbidities, *n* (%)			
Hyperlipidemia	8 (47.1)	16 (11.7)	0.0011
Endocrine disorder	3 (17.6)	9 (6.6)	0.13
GI disorder	3 (17.6)	19 (13.9)	0.72
Connective tissue or dysautonomia	1 (5.9)	15 (10.9)	1.00

LOF, loss-of-function; CADD, combined annotation-dependent depletion; AN, anorexia nervosa; MDD, major depressive disorder; OCD, obsessive–compulsive disorder; ADHD, attention deficit hyperactivity disorder; GI, gastrointestinal; BMI, body mass index; IQR, interquartile range. *Median (IQR); Mann–Whitney *U* test. All other *p*-values from Fisher exact test. All comparisons are descriptive; no Bonferroni correction applied (*n* = 17 carriers).

Carriers (*n* = 17) showed a substantially higher prevalence of hyperlipidemia vs. non-carriers (8/17, 47.1% vs. 16/137, 11.7%; OR 6.72, 95% CI 2.09–21.55, Fisher two-sided *p* = 0.0011), consistent with the known functional role of *PLIN4* in lipid storage. Carriers also trended toward earlier age at onset (Mann–Whitney *p* = 0.047) and lower BMI nadir (*p* = 0.051). AN subtype distribution did not differ. All comparisons are descriptive; given *n* = 17 carriers, inferential statistics are presented for hypothesis generation only.

*Median (IQR); Mann–Whitney *U* test was used for continuous variables, and Fisher exact test was used for categorical variables.

From a clinical perspective, it will be important to determine how *PLIN4* variants may contribute to the genetic architectures that underpin different phenotypes of AN. The most direct link is between alterations in body fat distribution and concerns about body weight and shape, which is a core feature of AN. Patients with *PLIN4* LOF variants preferentially store body fat in regions of the body, such asthe abdomen (33), that may exacerbate body image distress in patients with AN. Whether *PLIN4* variants contribute to the neuropsychiatric dimensions of AN remains uncertain. *PLIN4* is expressed predominantly in adipose tissue and skeletal muscle, with minimal expression in the adult brain (43), although neuronal expression has been reported in specific pathological contexts (44–46). Until recently, the physiological significance of LDs in central nervous system (CNS) neurons was considered uncertain (47); accumulating evidence now demonstrates that fatty acids derived from neuronal LD serve as an important metabolic fuel reserve sustaining synaptic transmission (48–50), raising the possibility that impaired LD-to-mitochondria fatty acid flux may compromise neuronal function. Whether *PLIN4* LOF contributes to this process warrants direct experimental investigation.

### Rare-variant landscape in AN

The present finding fits within an expanding rare-variant landscape in AN. Walker et al. (9) reported the largest CNV burden analysis to date and found no significant enrichment of rare CNVs across *n* = 7,414 AN cases, positioning rare point-mutation burden studies as the primary discovery paradigm for AN rare-variant biology. Verebi et al. (16) identified a familial AN pedigree harboring a novel *HCRTR1* splice donor variant (c.199 + 2T>G), providing a second contemporary candidate alongside the *BBOX1* (11) and *PLIN4* findings reported by our group. The convergence of independent rare-variant signals across orexin signaling, carnitine biosynthesis, and LD biology supports a model in which AN risk is mediated by multiple rare-variant loci affecting energy storage, mobilization, and CNS feeding circuits.

### Limitations

This study has several limitations. First, the cohort is a clinically ascertained single-center referral population enriched for treatment-resistant AN, predominantly of NFE ancestry, and is not a population sample of AN (see the “Cohort lineage” section). Carrier-frequency and burden estimates may therefore not generalize to population-ascertained cohorts.

Second, cases were ascertained from a single specialist eating-disorders clinic (Precision Psychiatry, Plano TX) while controls were drawn from gnomAD v4 NFE individuals. This introduces potential ascertainment bias: clinic-ascertained cases are enriched for severe and treatment-refractory AN, which may differ in genetic architecture from population-ascertained AN. The gold-standard within-cohort case/control design [e.g., *MC4R*-obesity in ALSPAC (31)] requires both cases and controls to be drawn from the same prospectively recruited population; our study does not achieve this. The *PLIN4* burden estimates should therefore be interpreted with this limitation in mind, and replication in a within-cohort design is the priority of follow-up work.

Third, although the burden *p*-value does not reach the threshold for genome-wide significance (*p* < 5e−08), that threshold is appropriate for unbiased GWAS scans rather than for a pre-specified candidate-gene panel; Bonferroni correction across 32 candidate genes is the appropriate standard for the present design.

Fourth, while predicted LOF variants in *PLIN4* are reported to have functional consequences on body fat distribution and lipid levels (33), the other putatively damaging variants reported as deleterious are based solely upon *in silico* prediction tools, which remains controversial (51). One previous GWAS reported an association of *PLIN4* and AN that did not reach genome-wide significance (*p* = 0.0012) (54), consistent with the possibility that both rare and common variants mediate risk of developing AN via *PLIN4*. In addition to GWAS and WES approaches, it will be important to use long-read sequencing methods to interrogate the role of *PLIN4* variants in AN. Because *PLIN4* contains a variable number tandem repeat in exon 3 made of a 99-base-pair motif, long-read sequencing is required to completely identify genetics in *PLIN4*; such complex repeat expansions cannot be reliably detected by standard short-read sequencing (52). Finally, future experiments will need to determine how the identified variants affect protein function.

### Replication plan

Independent replication of the *PLIN4* burden in an externally ascertained AN cohort is the priority of follow-up work. We have initiated outreach to the Genetics of Eating Disorders Study at the Broad Institute (https://www.broadinstitute.org/EDgenetics), which has a goal of recruiting 10,000 patients with eating disorders and 10,000 control subjects. The study’s use of blended exome genome sequencing is the ideal dataset for confirming our observations (53).

### Clinical implications

Routine clinical genetic testing for *PLIN4* variants in patients with AN is not currently warranted: the present finding requires replication in independent cohorts before guideline-level recommendations can be made, and the absolute increased risk associated with a single *PLIN4* LOF variant remains undetermined without family-level penetrance estimates. If a *PLIN4* LOF variant is identified incidentally in a child, current best practice would be familial cascade testing to identify additional carriers, behavioral and weight-trajectory monitoring during the high-risk adolescent window, and genetic counselling that emphasizes increased but non-deterministic risk. We would not recommend predictive genetic testing for *PLIN4* in asymptomatic individuals at this stage.

## Conclusions

Patients with AN carry a statistically significant excess of rare, putatively damaging variants in *PLIN4*. The combined CADD > 15 + LOF burden survives Bonferroni correction across all 32 candidate genes from the index pedigree screen; LOF-only carriers contribute independent co-segregation evidence across multiple pedigrees. These findings implicate LD biology in adipose tissue and skeletal muscle as a candidate contributor to AN susceptibility, warranting independent replication in externally ascertained cohorts.

## Data Availability

The original contributions presented in the study are included in the article/**Supplementary Material**, further inquiries can be directed to the corresponding author/s.
