# Supplementary material for: Increased frequency of rare damaging variants in PLIN4 in patients with anorexia nervosa
Source: Front Psychiatry. 2026 Oct 8;17:1937613. doi: 10.3389/fpsyt.2026.1937613 (PMC13687432; doi:10.3389/fpsyt.2026.1937613)
Supplement: Supplementary file 1 [file SupplementaryFile1.docx]

**Supplementary Table S1. Rare damaging variants identified in an individual with anorexia nervosa from the index family (CADD ≥ 15 or loss-of-function; gnomAD v4 NFE frequency < 0.01). All 44 variants across 32 candidate genes are listed. Chromosomal positions are GRCh38.**

| **Chr** | **Position** | **rsID** | **Ref** | **Alt** | **Gene** | **Variant type** | **AA change** | **gnomAD All** | **gnomAD NFE** | **CADD** | **GERP** | **REVEL** |
| --- | --- | --- | --- | --- | --- | --- | --- | --- | --- | --- | --- | --- |
| 1 | 32695140 | rs199898505 | C | T | *SYNC* | missense | p.E320K | 0.000331 | 5.52e-05 | 24.7 | 4.38 | 0.591 |
| 1 | 33096711 | rs145723160 | C | T | *AZIN2* | missense | p.A253V | 0.000221 | 5.85e-05 | 25.1 | 3.37 | 0.226 |
| 1 | 39412343 | rs147147341 | A | G | *KIAA0754* | missense | Q693R | 0.000271 | 5.93e-06 | 15.2 | . | 0.195 |
| 1 | 42850981 | rs145788428 | G | A | *ZNF691* | missense | p.S39N | 5.39e-05 | 3.48e-06 | 6.13 | . | 0.144 |
| 1 | 46799184 | rs55672106 | C | T | *CYP4B1* | missense | p.R35W | 0.000896 | 0.00039 | 15.6 | . | 0.375 |
| 1 | 47287615 | rs75426387 | C | T | *STIL* | missense | p.A357T | 0.000442 | 3.65e-05 | 12.4 | . | 0.035 |
| 1 | 52606760 | rs168820347 | G | A | *GPX7* | missense | p.R72Q | 0.000331 | 5.93e-06 | 23.7 | 3.21 | 0.085 |
| 1 | 86443872 | rs141677634 | T | C | *CLCA2* | missense | p.M525T | 0.002774 | 0.00085 | 3.05 | 2.18 | 0.048 |
| 2 | 112662996 | rs151199405 | A | G | *SLC20A1* | missense | p.I671V | 0.000264 | 0.00043 | 2.55 | . | 0.112 |
| 2 | 189677062 | rs141582205 | A | C | *ANKAR* | missense | p.X191T | 0.001737 | 0.00209 | 20.6 | . | 0.129 |
| 2 | 201880092 | rs145205966 | C | T | *CDK15* | missense | p.R375C | 0.000575 | 0.00051 | 26.9 | 6.06 | 0.631 |
| 3 | 32391967 | rs11717165 | G | C | *CMTM7* | missense | p.G21R | 0.003946 | 0.00457 | 15.9 | 2.56 | 0.071 |
| 3 | 171687409 | rs369537882 | T | C | *PLD1* | missense | p.H572R | 9.11e-05 | 0.00012 | 8.72 | . | 0.059 |
| 4 | 15502836 | rs186264635 | T | G | *CC2D2A* | missense | p.S117R | 0.001791 | 0.00213 | 22.6 | . | 0.104 |
| 6 | 6167600 | rs5983 | A | T | *F13A1* | missense | p.L589Q | 0.005122 | 0.00604 | 1.42 | . | 0.062 |
| 6 | 151573432 | rs55868409 | G | A | *CCDC170* | missense | p.E345K | 0.008318 | 0.01041 | 24.1 | 4.66 | 0.097 |
| 6 | 159907326 | rs142220585 | C | T | *MAS1* | missense | p.T124M | 0.000397 | 0.00047 | 24.7 | 5.65 | 0.583 |
| 6 | 169659165 | rs763294437 | C | T | *WDR27* | missense | p.V414M | 4.59e-05 | 4.75e-05 | 0.435 | . | 0.135 |
| 7 | 27130714 | rs753562339 | A | G | *HOXA4* | missense | p.I7S | 2.49e-06 | 2.55e-06 | 28.2 | 3.63 | 0.84 |
| 7 | 128396989 | rs72624961 | C | T | *IMPDH1* | missense | p.A370T | 0.003201 | 0.00404 | 22.3 | 5.19 | 0.222 |
| 7 | 134933628 | rs148403238 | A | C | *CALD1* | missense | p.K287Q | 0.00028 | 0.00033 | 13.9 | 2.15 | 0.039 |
| 8 | 8892410 | rs142747070 | G | A | *MFHAS1* | missense | p.R217W | 0.000239 | 0.00031 | 25.4 | . | 0.066 |
| 8 | 94170963 | rs111589116 | G | A | *CDH17* | missense | p.A269V | 0.000276 | 5.60e-05 | 22.6 | 5.95 | 0.207 |
| 8 | 94260392 | rs35262320 | G | A | *GEM* | missense | p.H38Y | 0.000548 | 8.1e-05 | 19.4 | 4.66 | 0.066 |
| 8 | 112337702 | rs368245391 | G | A | *CSMD3* | missense | p.R2228C | 1.10e-04 | 0.00013 | 27.4 | 5.64 | 0.515 |
| 9 | 18721620 | rs749548786 | C | T | *ADAMTSL1* | missense | p.P654L | 1.74e-05 | 7.63e-06 | 26.3 | 5.86 | 0.459 |
| 11 | 57301020 | rs139208640 | G | A | *TNKS1BP1* | missense | p.R1665C | 0.004856 | 0.00585 | 31 | 5.11 | 0.319 |
| 11 | 57302118 | rs199888470 | G | A | *TNKS1BP1* | missense | p.S1597L | 0.00015 | 0.00018 | 27.3 | 5.33 | 0.095 |
| 11 | 119018904 | rs143175328 | G | A | *TRAPPC4* | missense | p.D37N | 3.10e-06 | 4.24e-06 | 25 | 5.86 | 0.477 |
| 11 | 120962485 | rs150174191 | G | A | *GRIK4* | missense | p.M690I | 0.00139 | 0.00173 | 27.2 | 5.69 | 0.554 |
| 12 | 70555234 | rs61758735 | G | A | *PTPRB* | missense | p.T1690I | 0.006864 | 0.00819 | 25.2 | 4.86 | 0.113 |
| 12 | 95986106 | rs117991621 | C | T | *HAL* | missense | p.R369Q | 0.003752 | 0.00463 | 28.4 | 5.63 | 0.823 |
| 12 | 111598963 | rs749747800 | C | CTGTCTGCTGCC | *AXTN2* | nonframeshift ins | p.Q28_P29insQQQ | 5.46e-05 | 1.9e-05 | 5.89 | . | . |
| 14 | 21522208 | rs200715579 | T | G | *SALL2* | missense | p.M841L | 0.003333 | 0.00411 | 12.8 | . | 0.033 |
| 14 | 34539574 | rs370344686 | C | A | *EAPP* | stopgain (LOF) | p.E19X | 2.12e-05 | 2.38e-05 | LOF | 4.29 | . |
| 14 | 54963043 | rs753345230 | C | T | *WDHD1* | missense | p.E814K | 5.14e-05 | 6.53e-05 | 23.1 | 4.99 | 0.393 |
| 15 | 67208655 | rs200297750 | G | A | *AAGAB* | missense | p.P208S | 0.00024 | 0.00031 | 15.9 | . | 0.059 |
| 15 | 72407166 | rs754187366 | C | A | *TMEM202* | missense | p.L190I | 9.30e-06 | 1.10e-05 | 18.7 | 2.93 | 0.206 |
| 16 | 57724824 | rs114195136 | C | T | *DRC7* | missense | p.R583W | 0.006843 | 0.00572 | 25.2 | 2.99 | 0.255 |
| 16 | 71652887 | rs142472702 | C | T | *PHLPP2* | missense | p.R907Q | 0.000656 | 0.00085 | 28 | 5.41 | 0.485 |
| 17 | 3580491 | rs17633288 | T | C | *TRPV1* | missense | p.T505A | 0.00403 | 0.00436 | 8.64 | . | 0.363 |
| 17 | 5450213 | rs148615687 | C | T | *DHX33* | missense | p.G573D | 0.000198 | 0.00015 | 13.9 | 5.83 | 0.135 |
| 19 | 4512673 | rs780147097 | T | TAGGACAGTCTTACTGGTGTCCACGCC | *PLIN4* | frameshift ins (LOF) | p.Thr430AlafsTer9 | 3.55e-05 | 0.00012 | LOF | . | . |
| 20 | 34986970 | rs200924846 | A | G | *MYH7B* | missense | p.E372G | 0.007439 | 0.00948 | 25.4 | 3.66 | 0.401 |

*Chr = chromosome; Pos = GRCh38 position; Ref = reference allele; Alt = alternate allele; gnomAD All = gnomAD v4 all-population allele frequency; gnomAD NFE = gnomAD v4 non-Finnish European allele frequency; CADD = CADD v1.6 Phred score (LOF = loss-of-function variant); GERP = GERP++ conservation score; REVEL = REVEL pathogenicity score; ins = insertion; . = not available. Gene symbols in italics. EAPP stopgain (p.E19X) is carried by the index proband alongside the PLIN4 frameshift; EAPP burden is not significant after Bonferroni correction (0/153 carriers in the proband-removed analysis).*

**Supplementary Table S2. PLIN4 variant genotype data from AN case whole-exome sequencing. Loss-of-function variants (top section) and rare missense variants with CADD > 15 (bottom section). Chromosomal positions are GRCh38.**

**Loss-of-Function Variants**

| **rsID** | **Chromosomal position (GRCh38)** | **Predicted effect** | **Amino acid change** | **GT:AD:DP:GQ:PL** |
| --- | --- | --- | --- | --- |
| rs780147097 | 19-4512673-T-TAGGACAGTCTTACTGGTGTCCACGCC | LOF (frameshift ins) | p.Thr430AlafsTer9 | 0/1:105,23:128:99:685,0,4406 |
| rs780147097 | 19-4512673-T-TAGGACAGTCTTACTGGTGTCCACGCCGTCTG | LOF (frameshift ins) | p.Thr430ArgfsTer11 | 0/1:137,25:162:99:637,0,5658 |
| rs780147097 | 19-4512673-T-TAGGACAGTCTTACTGGTGTCCACGCCGTCTG | LOF (frameshift ins) | p.Thr430ArgfsTer11 | 0/1:70,8:78:99:242,0,3032 |
| rs534069616 | 19-4510708-C-CGGGG | LOF (frameshift ins) | p.Phe1089ProsfsTer32 | 0/1:194,119:313:99:4462,0,7880 |
| rs201581703 | 19-4512804-G-A | LOF (nonsense) | p.Gln386Ter | 0/1:101,95:196:99:2510,0,2765 |
| rs376558815 | 19-4504902-G-A | LOF (nonsense) | p.Gln1250Ter | 0/1:152,165:317:99:4512,0,4106 |

**Rare Missense Variants (CADD > 15)**

| **rsID** | **Chromosomal position (GRCh38)** | **CADD score** | **Amino acid change** | **GT:AD:DP:GQ:PL** |
| --- | --- | --- | --- | --- |
| rs201218860 | 19-4511600-G-T | 22.2 | p.Thr787Asn | 0/1:72,54:126:99:1484,0,2219 |
| rs201219499 | 19-4510481-C-T | 18.5 | p.Gly1160Glu | 0/1:166,120:286:99:2896,0,4473 |
| rs201219499 | 19-4510481-C-T | 18.5 | p.Gly1160Glu | 0/1:183,199:382:99:5330,0,5027 |
| rs201219499 | 19-4510481-C-T | 18.5 | p.Gly1160Glu | 0/1:130,97:227:99:2234,0,3084 |
| rs374438181 | 19-4511754-C-T | 15.4 | p.Gly736Arg | 0/1:391,58:449:99:1024,0,10291 |
| . | 19-4512264-C-A | 16.1 | p.Lys566Asn | 0/1:112,92:204:99:2717,0,3259 |
| rs201410585 | 19-4508805-C-A | 22.8 | p.Arg1222Met | 0/1:60,66:126:99:1993,0,1715 |
| rs201390076 | 19-4512126-C-T | 23.6 | p.Ala612Thr | 0/1:135,116:251:99:3526,0,4132 |
| rs201390076 | 19-4512126-C-T | 23.6 | p.Ala612Thr | 0/1:141,173:314:99:5336,0,4002 |
| rs62115187 | 19-4511610-C-A | 22.4 | p.Gly784Cys | 0/1:99,25:124:99:530,0,4160 |
| rs62115187 | 19-4511610-C-A | 22.4 | p.Gly784Cys | 0/1:88,15:103:99:530,0,3680 |

*GT = genotype (0/1 = heterozygous carrier); AD = allelic depth (ref reads, alt reads); DP = total read depth; GQ = genotype quality (Phred-scaled); PL = phred-scaled genotype likelihoods (homRef, het, homAlt). ins = insertion. . = no dbSNP identifier assigned. Multiple rows for the same rsID indicate independent patients carrying the same variant.*

**Supplementary Table S3. Rare damaging variant burden across 32 candidate genes versus gnomAD v4 non-Finnish European controls, Bonferroni-corrected for 32 tests. Primary analysis: n = 153 AN cases (proband removed). Sensitivity analysis: n = 154 AN cases (proband retained). Genes sorted by one-sided Fisher p (primary analysis).**

**Primary analysis: proband removed (n = 153 AN cases)**

| **Gene** | **AN carriers / n (153)** | **gnomAD v4 NFE carriers / denom** | **OR (95% CI)** | **Fisher p (1-sided)** | **Bonf p (1-sided)** | **Fisher p (2-sided)** | **Bonf p (2-sided)** | **Direction** |
| --- | --- | --- | --- | --- | --- | --- | --- | --- |
| *PLIN4* | 16 / 137 | 18,724 / 568,226 | 3.64 (2.18-6.07) | 3.52 × 10⁻⁵ | 0.001 | 3.52 × 10⁻⁵ | 0.001 | Enriched |
| *DRC7* | 7 / 146 | 13,977 / 575,731 | 2.11 (1.01-4.39) | 0.073 | 1.00 | 0.099 | 1.00 | - |
| *GRIK4* | 10 / 143 | 23,734 / 560,693 | 1.73 (0.92-3.23) | 0.095 | 1.00 | 0.145 | 1.00 | - |
| *MYH7B* | 30 / 123 | 91,719 / 495,278 | 1.33 (0.90-1.98) | 0.109 | 1.00 | 0.181 | 1.00 | - |
| *AAGAB* | 5 / 148 | 11,676 / 577,294 | 1.83 (0.78-4.29) | 0.189 | 1.00 | 0.235 | 1.00 | - |
| *ADAMTSL1* | 10 / 143 | 28,998 / 552,398 | 1.39 (0.74-2.61) | 0.235 | 1.00 | 0.351 | 1.00 | - |
| *WDHD1* | 6 / 147 | 16,326 / 566,929 | 1.53 (0.70-3.36) | 0.259 | 1.00 | 0.329 | 1.00 | - |
| *CYP4B1* | 2 / 151 | 5,965 / 583,631 | 1.61 (0.46-5.64) | 0.459 | 1.00 | 0.670 | 1.00 | - |
| *TNKS1BP1* | 12 / 141 | 46,742 / 536,575 | 1.01 (0.57-1.81) | 0.574 | 1.00 | 1.00 | 1.00 | - |
| *AZIN2* | 2 / 151 | 7,826 / 580,355 | 1.22 (0.35-4.27) | 0.605 | 1.00 | 1.00 | 1.00 | - |
| *IMPDH1* | 4 / 149 | 17,153 / 564,158 | 0.99 (0.39-2.53) | 0.664 | 1.00 | 1.00 | 1.00 | - |
| *CDH17* | 2 / 151 | 8,914 / 577,173 | 1.07 (0.31-3.73) | 0.678 | 1.00 | 1.00 | 1.00 | - |
| *PHLPP2* | 5 / 148 | 23,460 / 559,811 | 0.88 (0.38-2.07) | 0.741 | 1.00 | 0.836 | 1.00 | - |
| *CSMD3* | 9 / 144 | 43,220 / 543,770 | 0.83 (0.43-1.59) | 0.801 | 1.00 | 0.641 | 1.00 | - |
| *PTPRB* | 8 / 145 | 39,284 / 548,855 | 0.82 (0.41-1.63) | 0.808 | 1.00 | 0.626 | 1.00 | - |
| *HAL* | 5 / 148 | 27,144 / 557,748 | 0.76 (0.32-1.78) | 0.842 | 1.00 | 0.563 | 1.00 | - |
| *CCDC170* | 4 / 149 | 24,316 / 564,612 | 0.70 (0.27-1.79) | 0.880 | 1.00 | 0.538 | 1.00 | - |
| *CDK15* | 1 / 152 | 8,343 / 580,967 | 0.68 (0.14-3.42) | 0.887 | 1.00 | 0.729 | 1.00 | - |
| *ANKAR* | 6 / 147 | 34,618 / 550,978 | 0.70 (0.32-1.54) | 0.894 | 1.00 | 0.390 | 1.00 | - |
| *TMEM202* | 1 / 152 | 8,573 / 578,402 | 0.66 (0.13-3.31) | 0.895 | 1.00 | 0.731 | 1.00 | - |
| *CC2D2A* | 4 / 149 | 27,526 / 555,535 | 0.61 (0.24-1.55) | 0.934 | 1.00 | 0.335 | 1.00 | - |
| *MFHAS1* | 1 / 152 | 19,293 / 568,007 | 0.29 (0.06-1.45) | 0.994 | 1.00 | 0.068 | 1.00 | - |
| *KIAA0754* | 13 / 140 | 107,226 / 474,871 | 0.43 (0.24-0.74) | 1.000 | 1.00 | 7.88 × 10⁻⁴ | 0.025 | Depleted |
| *CMTM7* | 0 / 153 | 5,199 / 554,161 | 0.35 (0.02-5.58) | 1.00 | 1.00 | 0.411 | 1.00 | - |
| *GEM* | 0 / 153 | 3,840 / 583,843 | 0.50 (0.03-7.95) | 1.00 | 1.00 | 0.631 | 1.00 | - |
| *GPX7* | 0 / 153 | 1,967 / 584,257 | 0.97 (0.06-15.54) | 1.00 | 1.00 | 1.00 | 1.00 | - |
| *SYNC* | 0 / 153 | 13,372 / 560,975 | 0.14 (0.01-2.19) | 1.00 | 1.00 | 0.055 | 1.00 | - |
| *MAS1* | 0 / 153 | 7,698 / 582,040 | 0.25 (0.02-3.96) | 1.00 | 1.00 | 0.275 | 1.00 | - |
| *TRAPPC4* | 0 / 153 | 2,320 / 587,590 | 0.82 (0.05-13.25) | 1.00 | 1.00 | 1.00 | 1.00 | - |
| *HOXA4* | 0 / 153 | 5,533 / 555,070 | 0.33 (0.02-5.25) | 1.00 | 1.00 | 0.412 | 1.00 | - |
| *EAPP* | 0 / 153 | 5,761 / 580,514 | 0.33 (0.02-5.27) | 1.00 | 1.00 | 0.412 | 1.00 | - |

**Sensitivity analysis: proband retained (n = 154 AN cases)**

| **Gene** | **AN carriers / n (154)** | **gnomAD v4 NFE carriers / denom** | **OR (95% CI)** | **Fisher p (1-sided)** | **Bonf p (1-sided)** | **Fisher p (2-sided)** | **Bonf p (2-sided)** | **Direction** |
| --- | --- | --- | --- | --- | --- | --- | --- | --- |
| *PLIN4* | 17 / 137 | 18,724 / 568,226 | 3.86 (2.35-6.35) | 9.98 × 10⁻⁶ | 3.19 × 10⁻⁴ | 9.98 × 10⁻⁶ | 3.19 × 10⁻⁴ | Enriched |
| *DRC7* | 7 / 147 | 13,977 / 575,731 | 2.09 (1.01-4.36) | 0.075 | 1.00 | 0.100 | 1.00 | - |
| *GRIK4* | 10 / 144 | 23,734 / 560,693 | 1.72 (0.92-3.21) | 0.098 | 1.00 | 0.146 | 1.00 | - |
| *MYH7B* | 30 / 124 | 91,719 / 495,278 | 1.32 (0.89-1.97) | 0.116 | 1.00 | 0.184 | 1.00 | - |
| *AAGAB* | 5 / 149 | 11,676 / 577,294 | 1.82 (0.78-4.26) | 0.192 | 1.00 | 0.238 | 1.00 | - |
| *ADAMTSL1* | 10 / 144 | 28,998 / 552,398 | 1.38 (0.74-2.59) | 0.241 | 1.00 | 0.354 | 1.00 | - |
| *WDHD1* | 6 / 148 | 16,326 / 566,929 | 1.52 (0.69-3.33) | 0.264 | 1.00 | 0.332 | 1.00 | - |
| *CYP4B1* | 2 / 152 | 5,965 / 583,631 | 1.60 (0.46-5.60) | 0.462 | 1.00 | 0.671 | 1.00 | - |
| *TNKS1BP1* | 12 / 142 | 46,742 / 536,575 | 1.01 (0.56-1.80) | 0.583 | 1.00 | 1.00 | 1.00 | - |
| *AZIN2* | 2 / 152 | 7,826 / 580,355 | 1.22 (0.35-4.24) | 0.609 | 1.00 | 1.00 | 1.00 | - |
| *IMPDH1* | 4 / 150 | 17,153 / 564,158 | 0.98 (0.38-2.51) | 0.669 | 1.00 | 1.00 | 1.00 | - |
| *CDH17* | 2 / 152 | 8,914 / 577,173 | 1.06 (0.30-3.70) | 0.681 | 1.00 | 1.00 | 1.00 | - |
| *PHLPP2* | 5 / 149 | 23,460 / 559,811 | 0.88 (0.37-2.06) | 0.746 | 1.00 | 0.837 | 1.00 | - |
| *EAPP* | 1 / 153 | 5,761 / 580,514 | 0.98 (0.20-4.92) | 0.781 | 1.00 | 1.00 | 1.00 | - |
| *CSMD3* | 9 / 145 | 43,220 / 543,770 | 0.82 (0.43-1.58) | 0.807 | 1.00 | 0.642 | 1.00 | - |
| *PTPRB* | 8 / 146 | 39,284 / 548,855 | 0.81 (0.41-1.62) | 0.814 | 1.00 | 0.626 | 1.00 | - |
| *HAL* | 5 / 149 | 27,144 / 557,748 | 0.76 (0.32-1.77) | 0.846 | 1.00 | 0.564 | 1.00 | - |
| *CCDC170* | 4 / 150 | 24,316 / 564,612 | 0.69 (0.27-1.77) | 0.883 | 1.00 | 0.539 | 1.00 | - |
| *CDK15* | 1 / 153 | 8,343 / 580,967 | 0.68 (0.14-3.40) | 0.889 | 1.00 | 0.730 | 1.00 | - |
| *TMEM202* | 1 / 153 | 8,573 / 578,402 | 0.66 (0.13-3.29) | 0.896 | 1.00 | 0.731 | 1.00 | - |
| *ANKAR* | 6 / 148 | 34,618 / 550,978 | 0.70 (0.32-1.53) | 0.898 | 1.00 | 0.390 | 1.00 | - |
| *CC2D2A* | 4 / 150 | 27,526 / 555,535 | 0.60 (0.24-1.54) | 0.936 | 1.00 | 0.257 | 1.00 | - |
| *MFHAS1* | 1 / 153 | 19,293 / 568,007 | 0.29 (0.06-1.44) | 0.994 | 1.00 | 0.068 | 1.00 | - |
| *KIAA0754* | 13 / 141 | 107,226 / 474,871 | 0.42 (0.24-0.74) | 1.000 | 1.00 | 7.93 × 10⁻⁴ | 0.025 | Depleted |
| *CMTM7* | 0 / 154 | 5,199 / 554,161 | 0.34 (0.02-5.54) | 1.00 | 1.00 | 0.411 | 1.00 | - |
| *GEM* | 0 / 154 | 3,840 / 583,843 | 0.49 (0.03-7.90) | 1.00 | 1.00 | 0.631 | 1.00 | - |
| *GPX7* | 0 / 154 | 1,967 / 584,257 | 0.96 (0.06-15.44) | 1.00 | 1.00 | 1.00 | 1.00 | - |
| *SYNC* | 0 / 154 | 13,372 / 560,975 | 0.14 (0.01-2.18) | 1.00 | 1.00 | 0.055 | 1.00 | - |
| *MAS1* | 0 / 154 | 7,698 / 582,040 | 0.24 (0.02-3.93) | 1.00 | 1.00 | 0.276 | 1.00 | - |
| *TRAPPC4* | 0 / 154 | 2,320 / 587,590 | 0.82 (0.05-13.16) | 1.00 | 1.00 | 1.00 | 1.00 | - |
| *HOXA4* | 0 / 154 | 5,533 / 555,070 | 0.32 (0.02-5.21) | 1.00 | 1.00 | 0.413 | 1.00 | - |
| *EAPP* | 0 / 154 | 5,761 / 580,514 | 0.33 (0.02-5.27) | 1.00 | 1.00 | 0.412 | 1.00 | - |

*a = AN carriers; b = AN non-carriers; c = gnomAD v4 NFE heterozygous carrier alleles; denom = per-gene representative AN/2 from gnomAD v4 NFE exomes (max AN/2 across variant sites). OR = odds ratio with Haldane-Anscombe correction (+0.5 to all cells); 95% CI from Wald log-OR SE. Fisher p (1-sided): pre-specified enrichment alternative (greater). Fisher p (2-sided): used to screen for depletion. Bonferroni denominator = 32 candidate genes. Genes with Bonferroni-corrected p < 0.05 (either direction) shown in bold: PLIN4 (enriched) and KIAA0754 (depleted). EAPP row appears twice: 1 carrier in n = 154 (proband is co-carrier); 0 carriers in n = 153 (proband removed).*
